# Microbial Electrochemical Technologies for Sustainable Nitrogen Removal in Marine and Coastal Environments

**DOI:** 10.3390/ijerph19042411

**Published:** 2022-02-19

**Authors:** María José De La Fuente, Carlos Gallardo-Bustos, Rodrigo De la Iglesia, Ignacio T. Vargas

**Affiliations:** 1 Departamento de Ingeniería Hidráulica y Ambiental, Facultad de Ingeniería, Pontificia Universidad Católica de Chile, Santiago 7820436, Chile; mjdelafu@uc.cl (M.J.D.L.F.); cugallardo@uc.cl (C.G.B.); 2 Marine Energy Research & Innovation Center (MERIC), Santiago 7550268, Chile; rdelaiglesia@bio.puc.cl; 3 Centro de Desarrollo Urbano Sustentable (CEDEUS), Santiago 7820436, Chile; 4 Departamento de Genética Molecular y Microbiología, Facultad de Ciencias Biológicas, Pontificia Universidad Católica de Chile, Santiago 7820436, Chile

**Keywords:** nitrogen removal, microbial electrochemical technologies (METs), bioelectrochemical reactors, nitrification, denitrification, seawater

## Abstract

For many years, the world’s coastal marine ecosystems have received industrial waste with high nitrogen concentrations, generating the eutrophication of these ecosystems. Different physicochemical-biological technologies have been developed to remove the nitrogen present in wastewater. However, conventional technologies have high operating costs and excessive production of brines or sludge which compromise the sustainability of the treatment. Microbial electrochemical technologies (METs) have begun to gain attention due to their cost-efficiency in removing nitrogen and organic matter using the metabolic capacity of microorganisms. This article combines a critical review of the environmental problems associated with the discharge of the excess nitrogen and the biological processes involved in its biogeochemical cycle; with a comparative analysis of conventional treatment technologies and METs especially designed for nitrogen removal. Finally, current METs limitations and perspectives as a sustainable nitrogen treatment alternative and efficient microbial enrichment techniques are included.

## 1. Introduction

### 1.1. Nitrogen Cycle Disturbance: A Big Silent Problem

The most commonly discussed global problem is climate change, but there is an even more substantial silent problem: nitrogen cycle alteration. Rockström et al. [1] proposed nine processes that regulate the Earth system’s stability and resistance, including climate change, entry of novel entities, stratospheric ozone depletion, atmospheric aerosol loading, ocean acidification, freshwater use, land system change, biosphere integrity, and biogeochemical flows of nutrients.

Within these processes, the most altered process that compromises sustainable development is the biochemical flow of nutrients (i.e., nitrogen and phosphorus). It is postulated that exceeding these limits increases the probabilities of abrupt and even irreversible changes [1].

One significant impact that population growth has generated globally is accelerating nitrogen entry rates into the biosphere [1,2]. The maximum global annual nitrogen entry rate (i.e., annual nitrogen deposition limit) has been calculated at 35 million tons of nitrogen, and today, the yearly nitrogen deposition is close to 150 million tons [1,3]. In this context, projections have been made, and the results only show that this alteration in the nitrogen deposition rate, which is altering the nitrogen cycle, will continue to increase [4,5]. Thus, alteration of the nitrogen cycle is a problem that should be addressed as a priority worldwide.

### 1.2. Sources and Effects of Nitrogen Excess on Coastal Marine Ecosystems

It has been determined that more than 40% of coastal marine ecosystems are impacted by anthropogenic activity, especially pollution by nutrients such as nitrogen [6,7,8]. Nitrogen can come from different anthropogenic sources, such as agriculture, livestock, industrial waste, and wastewater [9]. However, it has been identified that the anthropogenic sources with the most significant oceanic nitrogen discharge are submarine sewage outfalls and fish farming [10,11,12]. Historically, in submarine sewage outfalls (SSOs), all the wastewater went into the sea without nutrient and dissolved organic matter removal. An SSO usually includes primary treatment to remove the most significant solids (i.e., screening and sedimentation for gravity) and to inactivate bacteria (e.g., chlorination) [11,12]. According to Roberts et al. [13], a well-designed SSO does not require secondary or tertiary treatment, relying on its diffuser capacity. A proper diffuser should achieve an approximate dilution of 100:1 in deep outfalls. Due to the infrastructure costs, outfalls without these treatments have been widely used in developing countries. It has been estimated that the cost associated with secondary treatment is ten times the cost of primary treatment [13]. Nevertheless, contaminants of emerging concern (i.e., endocrine-disrupting chemicals and pharmaceutically active chemicals) not removed by primary treatment processes have been found near SSOs affecting aquatic organisms [14]. Indeed, in the Spanish Mediterranean coast, it has been reported that SSOs, including secondary treatment units, have less impact on local benthic communities than SSOs without this process [15].

For example, SSOs in Chile are widely used as a treatment system, in which approximately 29% of wastewater is transported directly to the sea through SSOs, corresponding to 254 MM m^3^ yr^−1^ [16]. The significant marine dilution and the pressure applied to the discharged water allow submarine outfalls to reduce pollutant concentrations that were not eliminated and comply with environmental standards [17,18]. Consequently, SSO technology does not remove dissolved carbon, nitrogen, or phosphorus, the primary pollutants in wastewater, affecting marine sediments. Recently, Gomez et al. [19] found that in tropical zones such as the Taganga Bay in Colombia, the additional nutrients and organic matter inputs from SSO affect macrofauna communities in a cascading effect coupled with natural upwelling. Higher temperatures affecting oxygen solubility could lead to anoxic zones due to the increased microbial respiration for excess organic matter. This condition could end in an increment of sulfide generation, a suppression of denitrification, and an increase in ammonification. These phenomena stimulate the growth of algae leading to eutrophication [19].

The fish farming industry has sustained growth since 1980 [20]. Marine aquaculture production of fish, shellfish, and seaweed in Chile reached 1.4 million tons in 2019, making it the leading mariculture producer in the western world [21]. This industry’s primary wastes are uneaten food, fish feces, and urea, contributing significantly to nutrient pollution in adjacent marine ecosystems [20]. These wastes are particularly rich in nitrogen (ammonia and nitrite), which generates an excess of the system’s load capacity, leading to a deterioration in water quality and even eutrophication of the ecosystem [20,22,23]. Today, nitrogen treatment options in the fish farming industry of developing countries, such as Chile, are only based on controlling the intensity of fish production and removing suspended matter using sedimentation basins and rotating drums filters [24,25,26,27]. However, in the last years and due to technological advances in the field, recirculating aquaculture systems (RAS) for massive marine farming are gaining more attention and interest. RAS have the potential of reduced water and energy consumption in a stable cultivation environment.

In developed countries with an active aquaculture industry (e.g., Norway, The Netherlands, Denmark), the development and application of RAS have experienced a significant advance. During the last two decades, research on RAS has ended in developing different designs for indoor and outdoor production [28]. RAS have been indicated as a feasible solution to avoid eutrophication derived from the fish farming industry [29]. However, there is no single recommended design for RAS in the industry. One of the critical problems in RAS is related to the load and accumulation of suspended solids, in particular, biogenic colloidal particles. Yogev and Gross [30] estimated that RAS coupled with modern treatment technologies might be able to reduce about 89%, 69%, and 100% of total ammonia nitrogen, nitrite, and nitrate, respectively [29].

Fish farming and urban wastewater discharge zones have shown concentrations from 12.48 to 40.67 mg L^−1^ for nitrate and from 5.29 to 54.90 mg L^−1^ for ammonium [11,31,32,33,34]. These measurements are 20 to 70 times and 40 to 3000 times higher than the nitrate and ammonium averages in the non-disturbed coastal sea, respectively [35,36]. These high nitrogen concentrations can generate the eutrophication of marine systems adjacent to fish farming or SSO areas. The main environmental effects of eutrophication are: (i) the increase in suspended particles due to macroalgae blooms, leading to a decrease in light penetration, and (ii) the change in biomass of primary producers [37].

**Figure 1:**
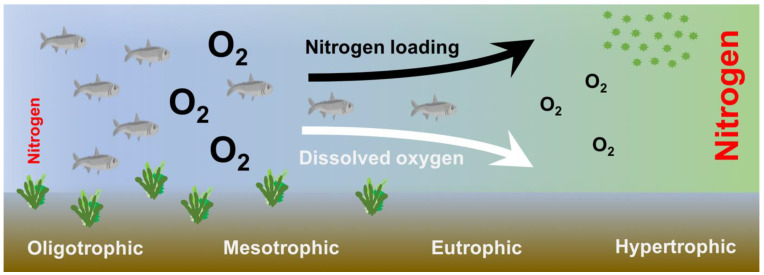
Conceptual schematic of marine eutrophication due to the increased loading of nitrogen (Modified from [37]).

Another significant effect is hypoxia caused by the decrease in dissolved oxygen. The production of CO_2_ associated with the decomposition of organic matter produced by the increase in primary productivity and suspended algae generates an increase in anoxic areas [8]. Eutrophication is usually followed by the proliferation of microalgae that are generally harmful and cause adverse effects on aquatic animals, such as obstruction of fish gills and localized anoxia [38,39] (Figure 1). In Chile, harmful algae blooms like *Pseudo-nitzschia australis*, *Pseudo-nitzschia calliantha*, *Protoceratium reticulatum*, and *Alexandrium* species have increased in the last four decades due to the eutrophication associated with the aquaculture industry. This algae proliferation has ended in different fish and shellfish-consuming related disease outbreaks, such as amnesic shellfish poisoning, paralytic shellfish poisoning, diarrhetic shellfish poisoning, and yessotoxins-related diseases [40].

### 1.3. Microorganisms Responsible for the Natural Metabolization of Nitrogen

**Figure 2:**
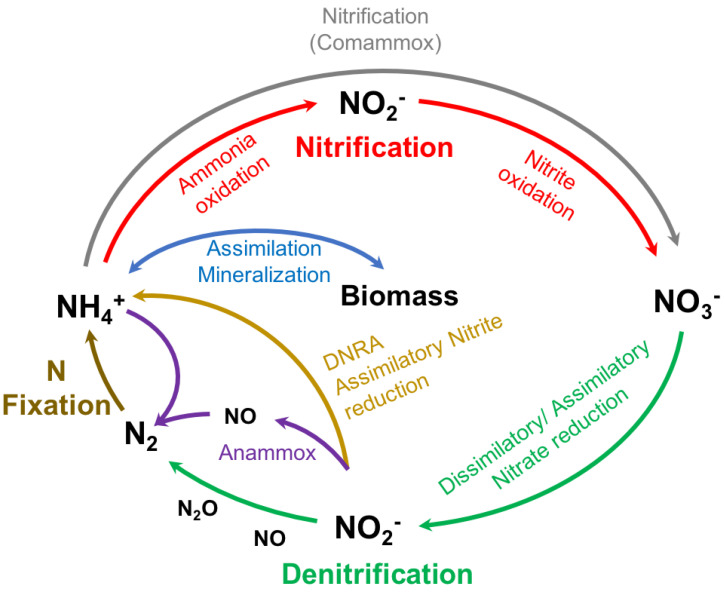
Biogeochemical cycle of nitrogen. Schematic illustration of the key processes of the nitrogen cycle (adapted from [45]).

The nitrogen cycle is carried out by a wide diversity of microorganisms responsible for maintaining the balance in nitrogen concentrations in coastal (and other) marine ecosystems. Nitrogen naturally enters marine systems through the nitrogen fixation process, transforming molecular nitrogen into ammonium (Figure 2). This process is carried out by N2-fixing microorganisms such as *Synechococcus* and *Trichodesmium* [41,42]. Then, ammonium oxidation to nitrite is carried out by ammonia-oxidizing bacteria (AOB), such as *Nitrosomonas* and *Nitrosospira*. Then, nitrite-oxidizing bacteria (NOB) (e.g., *Nitrobacter*, *Nitrococcus*, *Nitrospina*, and *Nitrospira*) oxidize nitrite to nitrate [43]. However, a few *Nitrospira* species can complete the nitrification process, transforming ammonium into nitrate. This last metabolism is called complete ammonia oxidation (COMAMMOX) [44,45]. In the next stage of microbial nitrogen metabolism, nitrate is reduced to molecular nitrogen by a broad group of microorganisms, denitrifying bacteria [42]. In addition to denitrification, another type of microbial metabolism removes nitrogen from the system by anaerobic ammonium oxidation (anammox). Indeed, it has been estimated that anammox contributes about 30 to 35% of the total nitrogen turnover in marine environments [46]. These bacteria, belonging to the *Planctomycetes* group, can metabolize nitrite and ammonium and transform them into nitrogen gas. This metabolism, like denitrification, is carried out under anaerobic conditions [42]. These bacterial metabolic processes are called dissimilative because microorganisms perform cellular oxidation-reduction processes to remove or provide electrons for cellular energetics.

On the other hand, nitrogen assimilation processes reduce nitrate to ammonium for cellular biosynthesis [42,45]. Under undisturbed conditions, these microorganisms can maintain the balance of nitrogen concentrations in the medium. The maximum assimilation rate of ammonium and nitrate by bacteria in the seawater has been previously determined, reaching a value of 1.96 × 10^−3^ mg L^−1^ day^−1^ [47]. Therefore, with nitrogen discharges to the ocean at concentrations from 12.48 to 40.67 mg L^−1^ for nitrate and 5.29 to 54.90 mg L^−1^ for ammonium [11,31,32,33,34], the natural cycle of microbial nitrogen metabolization is not sufficient to remove the excess of this compound in the medium. For this reason, it is necessary to find a way to remove this excess nitrogen to avoid eutrophication problems in coastal marine systems.

## 2. Technologies for Nitrogen Removal

Several technologies have been developed to remove the nitrogen present in wastewater and RAS. Today, physicochemical, chemical, and biological technologies are used to remove nitrogen from wastewater [48]. Table 1 shows physicochemical technologies conventionally used for nitrogen removal, including ion exchange (IE), reverse osmosis (RO), electrodialysis (ED), and adsorption by activated carbon [48,49]. Although these technologies have proven to be efficient in nitrogen removal, they present unsolved problems such as high chemical use, high brine production, high energy consumption, and high material and regeneration cost (Table 1) [49,50,51,52].

On the other hand, bacteria carry out biological treatments, which convert ammonium and nitrate to nitrogen gas. These methods do not present problems such as byproduct formation or brine production, and in general, the operational cost is lower than that of physicochemical and chemical techniques [48,49,53,54].

Bacteria of the nitrogen cycle carry out biological treatments. Under aerobic conditions, the microbial oxidation of ammonium to nitrate occurs, and then, under anaerobic conditions, the microbial reduction of nitrate to molecular nitrogen takes place [55,56]. The first limitation of biological treatments is the differences in the oxygen level in which each process occurs. These differences make it challenging to carry out both operations simultaneously. For the same reason, today, they are carried out separately. In the nitrification or COMAMMOX process, aeration is used to maintain an optimum oxygen concentration to ensure the sufficient presence of oxygen to oxidize ammonium to nitrite and then nitrite to nitrate. In the denitrification process, aeration is not needed, but an electron donor (organic or inorganic) is necessary to ensure nitrate reduction [56]. Adding an inorganic electron source is significantly less expensive than adding an organic electron source for denitrification. However, it is still considered a significant expense during the nitrogen removal process [57]. Similarly, the biological treatment of nitrogen generates an excessive production of sludge that, in the long term, ends up being a problem due to the need of its treatment, management, and final disposal [58].

Biofilm-based technologies have emerged as an excellent alternative to tackle sludge generation and increase biomass per bioreactor volume. A microbial biofilm can be defined as aggregates of microorganisms that accumulate to each other and/or to a surface/carrier. Microorganisms are enclosed in a matrix of extracellular polymeric substances that confers adhesion, structure, protection, and water and nutrients reservoir [59]. The development of a complex and thick (i.e., hundreds of microns) biofilm allows chemical gradient and the formation of oxic and anoxic zones [60]. Hence, this biological treatment configuration results in a high biomass concentration, achieving high carbon and nitrogen removal levels. Some examples of these biofilm-based technologies are: moving bed biofilms reactors (MBBRs) [61], rotating biological contactors (RBCs) [62], membrane biofilm reactors (MBfRs) [63], and sequencing batch reactors (SBRs) [64]. Nevertheless, all these technologies have disadvantages, such as high energy consumption for MBBRs, mechanical failures for RBCs, membrane biofouling for MBfRs, and granular disintegration for SBRs [60].

Based on the disadvantages of conventional and non-conventional nitrogen treatment technologies, other strategies, such as microbial electrochemical technologies (METs), have begun to receive more attention. Although these technologies were initially conceived to produce energy, taking advantage of microbial metabolism has proven to be a sustainable option for removing pollutants such as nitrogen compounds [65]. The difference between a MET and a traditional nutrient removal system resides in the opportunity to use the same chemical energy present in the waste for its treatment, reducing the operating energy expenses (e.g., aeration systems) [49].

## 3. Microbial Electrochemical Technologies (METs) for Sustainable Nitrogen Removal

### 3.1. Principles of METs

**Figure 3:**
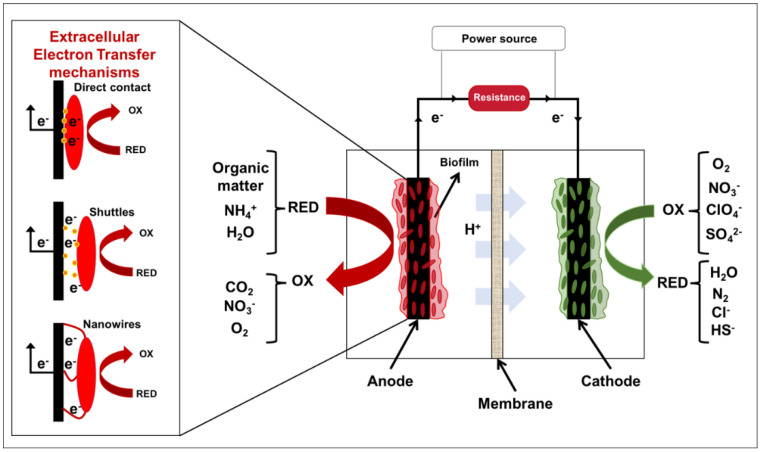
Illustration of the operation of a MET. OX: oxidized compounds, RED: compounds reduced (Modified from [68]).

METs are bioelectrochemical devices formed by an anode and a cathode connected through an external circuit, which allows the flow of electrons between the electrodes. A microbial biofilm grows on each electrode surface, catalyzes electron transfer in the system [73]. The operation of a MET is based on three fundamental stages (Figure 3). The first stage consists of the microbial oxidation of organic and inorganic compounds, thus managing to transfer electrons to the electrode to fulfill the role of the anode in the system [74]. This process is carried out by electrochemically active microorganisms (EAMs) capable of transferring electrons to the electrode [74,75]. During the second stage, a proton gradient occurs as a result of microbial metabolic processes. This is a critical point in the operation of a MET. If the proton concentrations increase excessively, the system’s pH will drop abruptly and prevent the growth and development of the microbial communities in the system [74]. This problem can be solved using seawater as the medium since it can counteract the increased production of protons [76]. Finally, the electrons transferred to the anode by the bacteria are transferred to the cathode, generating an electric current. In the cathode, biocathodic EAM catalyzes electrons’ transfer to a terminal electron acceptor such as nitrate. This process can also be chemically catalyzed (e.g., platinum), for example, for oxygen reduction [74].

An external power source has been used to impose a cell voltage, driving non-spontaneous chemical reactions, as hydrogen gas (H_2_) evolution [77] by assisting the electron flow through an external circuit, and then to promote microbial extracellular electron transfer (EET) due to the electrode polarization [78] (Figure 3). Since 2005, microbial electrolysis cells (MECs) have been studied to produce H_2_ in a cathode from the protons (H^+^) generated by the oxidation of organic (or inorganic) compounds in the anode (Figure 3). The applied voltage ranges from 0.45 V to 1.0 V, depending on the substrate and the MEC configuration [77,79]. In the last years, the same concept has been used to reduce anionic contaminants by a biocathode. For example, Torres-Rojas et al. [78] showed the reduction of chlorate by *Dechloromonas agitata* CKB in a bioelectrochemical reactor using an applied voltage of 0.44 V. In the same way, Zhan et al. [80] showed the influence of a low applied voltage (from 0.2 to 0.4 V) on nitrogen removal due to the enhance of autotrophic denitrification.

EAMs have specific mechanisms to capture or transfer electrons from or to an electrode. There are three known types of EET mechanisms in bacteria, outer membrane C-type cytochromes, shuttles, and nanowires [81] (Figure 3). C-type cytochromes work transferring electrons directly between the electrode and the bacteria. Therefore, this EET mechanism is proposed for bacteria directly contacting the electrode (i.e., anode and cathode). The shuttles are molecules secreted by bacteria to transfer electrons to the electrode. Studies carried out with Shewanella species suggest that mainly riboflavin and flavin mononucleotides can act as shuttles [82,83]. von Canstein and coworkers found a strong correlation between flavin mononucleotides secretion by Shewanella oneidensis and its growth due to the enhanced respiration of poorly soluble Fe(III) oxides in a culture medium [82]. Marsili et al. used electrochemical techniques to study the EET mediated by riboflavin and flavin mononucleotides. A significant decrease in current produced (i.e., 70%) by S. oneidensis reducing a carbon electrode was reported along with its restoration when the medium without planktonic cells was replaced. At the same time, cyclic voltammetry showed similar oxidation potential for electrodes colonized with S. oneidensis and electrodes incubated with riboflavin, suggesting that electron transfer was mediated by flavins [83]. Flavins have also been found to mediate EET in thick cell walls microorganisms, such as the Gram-positive bacteria Enterococcus faecalis ZER6 and Bacillus sp. WS-XY1 and yeast like Pichia stipitis [84,85]. While Zhang et al. reported an increase in current when glucose and riboflavins were added to the medium with *E*. *faecalis* [84], Wu et al. used differential pulse voltammetry, chronoamperometry, and high-performance liquid chromatography to elucidate the role of secreted flavins in the EET process of *P. stipitis* [85]. Other redox compounds widely distributed in environments or the extracellular polymeric substances present in biofilm (e.g., humic, manganese species, or polysulfide) could also be used as electron shuttles [86]. Phenazines (phenazine-1-carboxylic acid and pyocyanin) produced by Pseudomonas aeruginosa strains also mediate EET, enhancing its survival in anaerobic environments and improving MFC performance in mixed cultures [87,88,89,90]. Therefore, the bacterium does not need to be in direct contact with the electrode to interact with it. The third EET mechanism is through bacterial nanowires, which were found for the first time as electrically conductive pili in Geobacter sulfurreducens [91]. Bacterial nanowires can extend the contact with a solid electron acceptor or with other bacteria for about ten microns. Electrical conductivity has been demonstrated along nanowires [92], leaving bacteria within a biofilm to capture or transfer electrons to an electrode.

Extracellular electron uptake based on C-type cytochromes has been described for chemolithotrophic microorganisms forming biofilms on cathodes (i.e., biocathode), including microbial respiration of different terminal electron acceptors, such as oxygen, sulfate, chlorate, perchlorate, and nitrate [78,93,94,95,96]. To understand the metabolic pathways involved in these processes, further studies of new EAMs (i.e., different from *Geobacter* and *Shewanella*) able to remove these contaminants from water and soil are required.

### 3.2. MET as a Promising Nitrogen and Carbon Removal Strategy

Although MET was initially conceived to produce electrical energy taking advantage of the metabolism of EAM (i.e., microbial fuel cells), today, this family of technologies represents an energy-efficient and sustainable alternative for transforming different types of compounds. In this context, it has been possible to develop METs capable of removing carbon and nitrogen simultaneously [65]. Achieving the removal of carbon and nitrogen is a challenge mainly due to the microbial metabolism associated with the complete metabolization of nitrogen. As indicated in Section 1. 3 microbial nitrification occurs under strict aerobic conditions, while microbial denitrification occurs under anaerobic settings. This is why some of the first developed bioelectrochemical reactors (BERs) capable of removing carbon and nitrogen simultaneously separated nitrification from denitrification [97,98]. Since those early experiments, it was then discovered that denitrification could occur even with up to 5 mg L^−1^ oxygen concentrations in the medium [99]. This was possible due to the microbial stratification and the oxygen gradients generated in a biofilm, which would allow complete metabolization of nitrogen in the same biofilm [100,101,102,103,104]. These factors are due to the microbial stratification generated in the biofilm developed in the cathode. Although these reactors can simultaneously remove carbon and nitrogen, they still present challenges when applied in a complex system such as the sea [65,105]. These reactors have been developed using an inoculum from activated sludge from a reactor operating for several months or from previously enriched microbial consortia [104,105]. Using such inoculum ensures a significant abundance of nitrifying and denitrifying microorganisms in the reactor and its efficiency. The high physicochemical and biological complexity that characterizes the marine environment makes it impossible to ensure a high abundance of nitrifying and denitrifying microorganisms in a small volume [106]. This represents a considerable challenge to apply a MET capable of removing nitrogen and carbon in marine environments [107]

### 3.3. MET in Marine and Coastal Environments

METs have attracted attention as a sustainable bioremediation strategy to provide an alternative electron acceptor/donor in marine/coastal sediment. The redox gradient formed across a few centimeters of sediment allows the development of sediment-based METs. This approach has been named sediment microbial fuel cells (SMFC), benthic microbial fuel cells (BMFC), or microbial electrochemical snorkel (MES) [108,109,110,111]. In all of these variations, the anode electrode is buried in the sediment, providing an additional/external electron acceptor to chemoheterotrophic and chemoautotrophic microorganisms present in the sediment [108,109]. Following the same configuration as a conventional MFC (see Section 3.1), the external circuit connects the anode buried in an anoxic environment with a cathode, generally flowing in the oxygenated water column [112]. SMFCs and BMFCs have been proposed for in situ bioremediation and as alternatives to power wireless low-consuming sensors and biosensors [113,114]. MESs have been used for bioremediation in environments with excess organic matter, metals, and hydrocarbons [115,116,117]. Biocathodes on these types of METs can be used for different purposes, such as a catalyst for oxygen reduction replacing metallic catalysts and electron donor for oxidized species like sulfate, nitrite, and nitrate [108,109,118,119].

Bioelectrochemical denitrification in a SMFC has been studied for bioremediation of aquaculture streams using active biocathodes. Sajana et al. [120] compared COD removal and total Kjeldahl nitrogen (TKN) removal in sediments with and without cathode aeration. The authors tested different ambient temperatures (28–30 °C and 21–25 °C) and circuit configurations (100 ohms and short circuits). While higher temperatures and an aerated cathode were optimum for higher removal efficiencies, the short circuit mode reached an average COD and TKN removal efficiency of 84.4 ± 1.3% and 49.4 ± 8.3%, respectively, compared with the closed circuit (i.e., 100 ohms) condition that reached a COD and TKN removal of 79.4 ± 1.4% and 44.0 ± 5.4%, along with an average power density of 18.8 ± 1.3 (µW/m^2^) [120]. In a similar study, Sanders et al. [121] used an inverted scheme of anode/cathode configuration using electrolysis of water into oxygen in the anode as an electron source, reaching a denitrification rate of 0.3 ± 0.01 kg N/m^3^ d in saltwater [121].

Photosynthetic biocathodes using algal bacteria have also been tested as a nutrient removal strategy. The photosynthetic production of dissolved oxygen and dissolved organic matter in a cathode has shown power output improvements and enhanced nitrogen removal efficiency [122]. Sun et al. [123] reported an autonomous operation of a photo-bioelectrochemical system with day/night cycles. The system reached a maximum total nitrogen removal of 83% [123]. The authors explained the system’s high performance by the synergistic action of the produced photosynthetic oxygen used for nitrifying microorganisms, the photosynthetically produced dissolved organics used for heterotrophic denitrification, and the use of the cathode as an additional external electron donor for bioelectrochemical denitrification.

In terms of power generation and removal efficiencies, sediment-based METs in marine and coastal environments could be unstable in the short and long-term operation. This is because of their dependence on uncontrollable factors related to the environment itself, such as ambient temperature, pH, salinity, microorganisms, and minerals present in the sediment [109]. Using a SMFC, Kubota et al. [124] reported variations in power density outputs and sulfides removal in a eutrophicated coastal estuary in Tokyo. The fluctuation was due to weather conditions (i.e., cloudy vs. rainy days) and freshwater recharges [124]. Meanwhile, at long-term operation, Viggi et al. [117] reported the presence of non-conductive sulfide precipitates in the portion of the electrode immersed in the sediment, decreasing the active area in which the electroactive microorganisms could transfer electrons [117].

Controlling the development of specific microbial groups on anodes and cathodes is a difficult task in a conventional SMFC. Indeed, it has been reported that a marine environment permits the development of biofilms with a high diversity of microorganisms in the electrode. The electron acceptor/donor redundancy imposed by the electrodes and the interspecies electron transfer could drive a syntrophic coexistence of aerobic, anaerobic heterotrophic, and autotrophic microorganisms within bioanodes and biocathodes [125,126,127].

### 3.4. Electrochemical Overpotentials as a Microbial Enrichment Technique

In nature, there are microorganisms capable of using inorganic compounds as an electron source or final electron acceptors, such as iron-oxidizing bacteria and sulfate-reducing bacteria, respectively [128,129]. These cellular oxidation-reduction processes occur at specific electrochemical potentials (redox tower). Motivated by this, research has been carried out to determine the effect of polarization on microbial colonization of these electrodes [130,131,132,133,134]. Several authors have proven this strategy to modulate and enrich the presence of selected EAMs. An example of this is the research conducted by Torres et al. [134], where different overpotentials were applied to different electrodes. In this work, wastewater was used as an inoculum. As a result, different biofilms can be observed, depending on the overpotential applied to each electrode. When analyzing each electrode’s community composition, it was also possible to observe significant differences depending on the applied overpotential. [134]. Various authors have used this technique to enrich or promote specific microbial metabolism in an electrode [105,130,132,133]. Then, the electrochemical enrichment of specific metabolisms emerges as a novel and efficient strategy for biofilm modulation and control. This feature could be of particular interest in bioreactors operated under open environmental conditions by accelerating the startup process and improving their performance.

Using a microcosm approach, Rowe et al. [132] accomplished EAMs enrichment from marine sediments applying overpotentials from −50 to −400 mV (vs. Ag/AgCl). After incubation, the authors observed cathodic currents together with nitrate reduction, indicating denitrifying EAMs presence in marine sediments. Kondaveeti et al. [135] reported that the bioelectrochemical removal of nitrate shows biological reduction peaks at −130 mV and −260 mV (vs. Ag/AgCl) in a medium without and with nitrite, respectively. The authors also observed a reduction peak at −570 mV (vs. Ag/AgCl) if the medium contains only nitrite. Using three-electrode electrochemical cells and without the need for a culture medium, the addition of an exogenous electron donor, or a previously enriched inoculum, De La Fuente et al. [96] accomplished microbial enrichment of denitrifying microorganisms from natural seawater. It was done by applying the three potentials mentioned above associated with the dissimilatory denitrification process (i.e., −130, −260, and −570 mV vs. Ag/AgCl) [96]. The microbial community analysis conducted on biocathodes showed that applying −260 mV (vs. Ag/AgCl) to the working electrode made it possible to enrich Marinobacter and remove nitrate from water. To determine if this tool could contribute to the scalability and applicability of MET in natural environments, it is necessary to develop efforts for moving research from controlled laboratory conditions to complex natural settings, such as coastal marine environments.

As mentioned previously in this section, nitrification is usually accomplished in the anodic chamber (see Figure 3). Ammonia and nitrite oxidizers can be found as part of an anodic biofilm. For example, Zhan et al. [131] studied bioelectrochemical ammonium oxidation with the acclimation of BERs using an overpotential of +0.6 V (vs. Ag/AgCl). The authors observed a microbial community dominated by Nitrosomonas, Comamonas, and Paracocus. Similarly, Vilajeliu-Pons et al. [136] achieved anoxic nitrification with an imposed potential of +0.8 V vs. SHE, obtaining similar nitrogen removals compared with conventional treatments but 35 times lesser energy consumption.

On the other hand, Zhu et al. [137] enhance the anammox process in a bioanode, imposing an overpotential of −0.5 V (vs. Ag/AgCl) to facilitate the partial oxidation of ammonium to nitrite, compensating its lack in the reactor as an electron acceptor in the anammox process. However, in those systems, nitrification is usually not accomplished by nitrifying EAMs but by a diverse microbial community. Indeed, to the best of our knowledge, there is no experimentally proved evidence of the existence of this phenotype and its EET mechanism used to transfer electrons from reduced forms of nitrogen to the electrode (i.e., anode). Consequently, research on this topic is still in its infancy, and more efforts are required to thoroughly assess the use of specific overpotentials as a strategy to electrochemically enrich marine nitrifying microorganisms.

### 3.5. Future Opportunities for Applying MET in Coastal Environments

**Figure 4:**
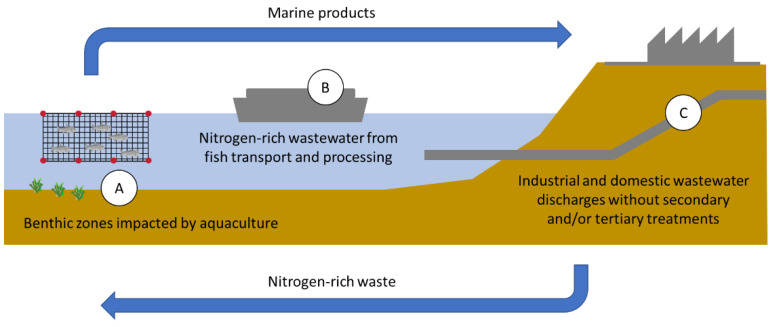
Visualized opportunities to develop METs for carbon and nitrogen removal in coastal and marine environments.

METs technology presents a series of advantages to be applied in multiple industrial processes in the future (Figure 4). It allows the effective removal of different compound types, and its electrochemical selection system will enable it to be adapted to the particular conditions of each treatment method. In the case of coastal systems, especially those associated with urban or industrial human settlements, METs have enormous potential. Associating this type of technologies as a previous treatment in SSOs would substantially reduce the nitrogen load of these wastewaters. However, scalability issues are primarily associated with the cost of electrode materials and chemical catalyzers. In addition, tailored reactor architecture is needed to reduce internal resistance, the formation of unwanted chemical gradients, and the loss of electrons to reactions that reduce efficiencies and generate by-products.

In the case of aquaculture systems, the industry could widely use this type of method to treat marine sediments under salmon fattening cages. In this way, anoxic beds would not be generated, which directly affects the quality of the environment. Furthermore, this methodology could be implemented in off-shore and coastal facilities without the need for large land space and high electric power requirements. Even more, the ships that transport dead fishes to coastal facilities would use METs to treat the wastewater generated in the process before being released into the sea.

## 4. Conclusions and Perspectives

Coastal systems have long been used as receptors for nitrogenous wastes, and there are currently many eutrophic coastal areas globally. A MET capable of removing carbon and nitrogen from marine environments could represent an alternative to prevent eutrophication problems in marine environments.

The potential scalability, cost-effectiveness relative to other technologies and the reviewed versatility and broad spectrum of configurations of these technologies led us to visualize, at least, three different areas of application in a coastal environment: (A) SMFCs used for in-situ remediation and monitoring of polluted benthic zones affected by fisheries; (B) off-grid autonomous BERs for treating high loads of carbon and nitrogen from dead fishes and wastewater generated during transportation; and (C) energy-friendly and low-cost wastewater modular treatment plants including carbon and nitrogen removal (Figure 4).

Improvements and previous analyses are needed before applying this technology in a natural marine system and achieving the desired impact. In addition, there is an open question of the existence of nitrifying EAMs. Further research is required to understand the microbial metabolic processes involved in EET of microorganisms associated with the nitrogen cycle. We visualize three points that should be addressed: (i) to find the optimal overpotential and time required to enrich microorganisms associated with the nitrogen cycle; (ii) to determine the biofilm-electrode’s surface area required to achieve bioelectrochemical nitrogen removal efficiency; and (iii) to isolate and study model denitrifying EAMs.

## Figures and Tables

**Table 1:** Comparison of different nitrogen removal techniques (modified from [48]).

Technology	Advantages	Disadvantages	References
Physicochemical			
Ion Exchange	Selective resins for different pollutants, common application, low production cost.	It requires the resin’s regeneration, brine production, and high use of chemicals (salt).	[50,66]
Reverse Osmosis	Remove multiple contaminants, low production cost, environmentally friendly.	Need for post-treatment to remove accumulated contaminants in brine, membrane fouling, high operating cost.	[51,67,68]
Electrodialysis	Multiple removals of pollutants, higher water recovery (less waste).	High energy consumption, complex construction, and operation skipping brine production as final waste.	[52,69]
Activated Carbon Absorption	It does not generate residues of brine or concentrates, high adsorption capacity, elimination of multiple contaminants.	High cost of material and the high price of regeneration.	[49,70]
Chemical			
Chemical denitrification.	Does not generate residues of brine or concentrates, nitrate reduction instead of accumulation in residues, elimination of multiple pollutants.	Inconsistency in nitrate reduction, pH, and temperature dependence. Risk of ammonia or nitrite production in the nitrate removal process.	[71,72]
Biological			
Conventional Biological nitrification and denitrification technologies.	No dangerous byproducts are generated, no additional treatment is required, removal of multiple pollutants, lower cost of operation than physicochemical treatments in general.	Constant oxygenation of the medium is necessary (nitrification), and the addition of organic or inorganic electron donor (denitrification) post-treatments is also required for turbidity and sludge removal.	[53,54]
Non-conventional biofilm-based technologies	High complex biomass concentration per volume of bioreactor. Chemical gradients coupled with oxygen gradient (oxic and anoxic zones) lead to increased carbon and nitrogen removal in the same compartment.	Possible high mass transfer resistance. Scaling-up problems such as biofouling, granular disintegration, and mechanical failures. It is highly affected by suspended solids.	[60,61]

## Data Availability

Not applicable.
